# Supplementary material for: Content-rich biological network constructed by mining PubMed abstracts
Source: BMC Bioinformatics. 2004 Oct 8;5:147. doi: 10.1186/1471-2105-5-147 (PMC528731; doi:10.1186/1471-2105-5-147)
Supplement: Additional File 2 — The original results of the above study (non-essential files are deleted to keep the file size under the limit set by BMC bioinformatics). [file 1471-2105-5-147-S2.bz2 › chilibotAdditionalFile2/dip20/27ID8999548E90/html/CASP1_IL18.html]

 


 **CASP1** and **IL18** 
  
Found 147 abstracts in PubMed, retrieved 20.  
 

 What does Google say? 
 PDF only 
| .edu only 

---

**Interactive relationship** (e.g. stimulation, inhibition, etc)

**Stimulatory relationship**- HBcAg dependent IL 18  [ **IL18** ]  levels were abrogated by inhibition of Caspase 1  [ **CASP1** ] , but not by blockade of CD40 CD154 interaction.  Ref: 12858406 J Med Virol, 2003
- Mice lacking caspase 1  [ **CASP1** ]  exhibited both improved peri infarct survival and a decreased rate of ventricular dilatation, possibly due in part to a decrease in MMP 3 activity, IL 18  [ **IL18** ]  production, and a reduction in the rate of apoptosis after experimental MI.  Ref: 12788386 J Mol Cell Cardiol, 2003
**Neutral relationship**- Recent publications have demonstrated that the protease caspase 1  [ **CASP1** ]  is responsible for the processing of pro interleukin 18  [ **IL18** ]  IL 18  [ **IL18** ]  into the active form.  Ref: 12874316 Infect ImmunInfect Immun, 2003
- In addition, splenocytes from IL 10 mice produced elevated levels of nitric oxide NO compared to WT mice, and NO has been shown to inhibit activity of interleukin 1 converting enzyme ICE  [ **CASP1** ] , which is required for IL 18  [ **IL18** ]  production.  Ref: 12670447 Cytokine, 2003
- When the cells were lysed in the presence of dithiothreitol, caspase 1  [ **CASP1** ]  precursor became active and converted pro IL 18  [ **IL18** ]  into mature IL 18  [ **IL18** ] .  Ref: 12706898 Gene, 2003
- Caspase 1  [ **CASP1** ]  is required for cleavage of the inactive precursor form of IL 18  [ **IL18** ]  into an active cytokine, and caspase 1  [ **CASP1** ]  deficient mice are resistant to lethal endotoxemia.  Ref: 12792854 J Infect Dis, 2003
- The release of IL 18  [ **IL18** ]  from mammalian cells is linked to its proteolytic activation by caspases including interleukin 1 converting enzyme ICE  [ **CASP1** ] .  Ref: 12597888 Protein Expr Purif, 2003
- As previously reported, transgenic mice KCASP1Tg, that over express caspase 1  [ **CASP1** ]  in their KC, display high serum levels of IgE, and spontaneously develop chronic dermatitis by production of IL 18  [ **IL18** ]  and IL 1beta.  Ref: 12697661 Int Immunol, 2003

**Non-interactive relationship** (e.g. studied together, co-existance, homology, etc.)

- Although IL beta plasma levels were not detectable, both IL 18  [ **IL18** ]  levels and the rate of apoptosis in remodeling, non infarcted muscle were significantly higher in WT compared to caspase 1  [ **CASP1** ]  KO animals.Conclusion.  Ref: 12788386 J Mol Cell Cardiol, 2003
- Taken together, these results indicate that HBcAg induces IL 18  [ **IL18** ]  secretion by induction of Caspase 1  [ **CASP1** ] .  Ref: 12858406 J Med Virol, 2003
- Caspase 1  [ **CASP1** ] , the prototypic caspase, is known to process the cytokines IL 1beta and IL 18  [ **IL18** ]  to mature forms but it is unclear whether, like other caspases, it can induce apoptosis by activation of downstream protease cascades.  Ref: 12444148 J Immunol, 2002
- To investigate the intra hepatic activation of the IFN system in patients affected by chronic HCV infection in comparison with that observed in a non infectious liver disease such as non alcoholic steatohepatitis, we measured the liver steady state mRNA levels of interferon alpha, interferon beta and interferon gamma as well as of IFN related genes IFNAR 1, STAT1alpha, PKR, 2 5 AS, IRF 1, ICE  [ **CASP1** ]  and IL 18  [ **IL18** ] .  Ref: 12794720 J Med Virol< MedlineTA>J Med Virol, 2002
- COS 7 cells transfected with plasmids encoding pro IL 18  [ **IL18** ]  and caspase 1  [ **CASP1** ]  precursor expressed the two proteins intracellularly.  Ref: 12706898 Gene, 2003
- Although IL 12 is also an IFN gamma inducing factor  [ **IL18** ] , the activity of IL 18  [ **IL18** ]  but not IL 12 in models of sepsis and death is dependent on the intracellular cysteine protease IL 1beta converting enzyme caspase 1  [ **CASP1** ] .  Ref: 12792854 J Infect Dis, 2003
- After MI, enhanced cardiac activity of the pro IL 18  [ **IL18** ]  processing enzyme, caspase 1  [ **CASP1** ] , was measured.  Ref: 12829183 Cardiovasc Res, 2003
- To produce an authentic IL 18  [ **IL18** ] , we have devised a bicistronic expression system for the coupled transcription and translation of ProIL 18 with caspase 1  [ **CASP1** ]  ICE  [ **CASP1** ]  or caspase 4 ICE  [ **CASP1** ]  rel II, TX, ICH 2.  Ref: 12597888 Protein Expr Purif, 2003
- Here, we report that hypoxic exposure of cultured brain microglia BV 2 mouse microglia cells and rat primary microglial cultures induces expression and activation of caspase 11, which is accompanied by activation of caspase 1  [ **CASP1** ]  and secretion of mature IL 1beta and IL 18  [ **IL18** ] .  Ref: 12829320 Brain Res Mol Brain Res, 2003
- Two recombinant hybrid baculoviruses containing cDNA of the porcine precursor IL 18  [ **IL18** ]  and the porcine caspase 1  [ **CASP1** ]  were constructed and were used to infect silkworm larvae.  Ref: 12655117 J Vet Med Sci, 2003
- We are reporting the molecular cloning of gerbil interleukin 18  [ **IL18** ]  IL 18  [ **IL18** ]  and caspase 1  [ **CASP1** ] .  Ref: 12706898 Gene, 2003
- The specific caspase 1  [ **CASP1** ]  inhibitor Ac YVAD CMK blocked the early IL 18  [ **IL18** ]  release in AM infected with the virulent strain.  Ref: 12874316 Infect ImmunInfect Immun, 2003
- While statins may stimulate the secretion of caspase 1  [ **CASP1** ] , IL 1beta and IL 18  [ **IL18** ]  in peripheral mononuclear cells in response to Mycobacterium tuberculosis, they exhibit additional effects on inflammation by decreasing IL 6 synthesis in human vascular smooth muscle cells VSMC in vitro.  Ref: 12698208 Drugs Today Barc , 2003
- Gerbil interleukin 18  [ **IL18** ]  and caspase 1  [ **CASP1** ]  cloning, expression and characterization.  Ref: 12706898 Gene, 2003
- Mice with targeted deletion of caspase 1  [ **CASP1** ]  interleukin 1beta IL 1beta converting enzyme lack the active forms of IL 1beta and IL 18  [ **IL18** ] , two cytokines implicated in maladaptive ventricular remodeling following cardiac injury.  Ref: 12788386 J Mol Cell Cardiol, 2003
